# Urinary Metabolites Reveal Hyperinsulinemia and Insulin Resistance in Polycystic Ovarian Syndrome (PCOS)

**DOI:** 10.3390/metabo11070437

**Published:** 2021-07-02

**Authors:** Anna Maria Fulghesu, Cristina Piras, Angelica Dessì, Claudia Succu, Luigi Atzori, Roberta Pintus, Cecilia Gentile, Stefano Angioni, Vassilios Fanos

**Affiliations:** 1 Clinica Ostetrica Ginecologica, Department of Surgical Science, Duilio Casula Hospital, University of Cagliari, SS 554, km 4.5, 09042 Monserrato, Italy; claudia.succu@gmail.com (C.S.); cecige@hotmail.it (C.G.); sangioni@yahoo.it (S.A.); 2 Department of Biomedical Sciences, University of Cagliari, SS 554, km 4.5, 09042 Monserrato, Italy; cristina.piras@unica.it (C.P.); latzori@unica.it (L.A.); 3 Neonatal Intensive Care Unit, Neonatal Pathology and Neonatal Section, University of Cagliari, SS 554, km 4.5, 09042 Monserrato, Italy; angelicadessi@unica.it (A.D.); gomberta@icloud.com (R.P.); vafanos@tiscali.it (V.F.)

**Keywords:** PCOS, insulin resistance, hyperinsulinemia, urine metabolomics

## Abstract

The identification of insulin resistance and hyperinsulinemia in polycystic ovary syndrome (PCOS) is not a minor issue. The homeostasis model assessment of insulin resistance index (HOMA) is the most used index of IR (Insulin Resistance), validated in overweight and obese patients but not in normal-weight PCOS subjects, who can still present with increased insulin secretion by an oral glucose tolerance test (OGTT). The evaluation of insulin secretion and resistance represents a still unresolved problem. The aim of this study is to identify a possible yet noninvasive method to properly evaluate the insulin metabolism in young non-diabetic subjects. Girls aged 14–22 years, afferent to the center of Gynecological Diseases in Childhood and Adolescence of Cagliari (Italy), were screened for PCOS. A total of 42 subjects comprised the study group. Hormonal assays, OGTT, transabdominal (TA) or transvaginal (TV) US, and urine collection for ^1^H-NMR analysis were assayed in the early follicular phase. A ^1^H-NMR coupled multivariate statistical analysis was performed. The OPLS model indicated that the NMR profile of urine had a good fit and prediction ability for the AUC OGTT with R^2^ = 0.813. Metabolomics can be a promising tool to the potential identification of biomarkers of an exaggerated insulin response to OGTT and can encourage substantial progress for a more accurate and early diagnosis in PCOS.

## 1. Introduction

Polycystic ovary syndrome (PCOS) is an endocrine and metabolic disease that affects 6% to 15% of women of reproductive age, according to the population considered and the diagnostic criteria applied [1,2].

PCOS is a multifactorial disorder characterized by genetic, endocrine, and metabolic abnormalities contributing to its development [3,4]. In addition, the heterogeneity in the clinical aspects of PCOS results in significant overlap of phenotypes, creating difficult challenges to identify the underlying causes [5,6]. Commonly, women with PCOS present clinical and hormonal hyperandrogenism (HA), chronic anovulation, and polycystic ovarian features on an ultrasound. Moreover, PCOS subjects are predisposed to type 2 diabetes, obesity, and cardiovascular disease [7,8], and often hyperinsulinemia and insulin resistance (IR) [9,10,11,12]. Recent studies have demonstrated that the principal endocrine features of PCOS are hyperandrogenemia and insulin resistance, independent from obesity [10,13,14]. Several symptoms of PCOS are boosted by hyperinsulinemia; a rise in insulin levels appears to promote androgen ovarian secretion, action, and an increase in weight.

The onset of the syndrome is the postmenarchal age, with appearances of hyperandrogenic and metabolic symptoms, which may severely affect the quality of life of these girls.

Obesity is a known source of hyperinsulinemia and insulin resistance (IR). Whereas, the presence of peripheral IR is ascertained in obese PCOS subjects [13], contrasting data exists on the increase of peripheral insulin resistance in normal-weight PCOS. Primary hyperinsulinemia can induce compensatory increases of peripheral insulin resistance [15]. The gold standard to diagnose IR is the euglycemic-hyperinsulinemic clamp [16].

The first report on a mild increase of IR in nonobese PCOS [17] was highlighted by others authors [18,19,20,21].

Many authors consider the HOMA Index (homeostasis model assessment of insulin resistance), which identifies fasting glycemic and insulinemic values, as a satisfactory surrogate to the clamp, because the clamp cannot be performed in daily practice due to its complexity, high cost, and discomfort to the patient. The HOMA offers results strongly correlated with the clamp in overweight and obese patients [22,23,24], but not all the studies are confirmatory in normal-weight PCOS subjects [25]. Insulin secretion is commonly evaluated assessing circulating C-peptide (C-pep) and insulin levels after glucose load through an oral glucose tolerance test (OGTT). An excessive insulin secretion after OGTT was demonstrated by several authors in 20–40% of lean PCOS individuals, independently from accompanying peripheral IR [9,26,27].

A study in 1997 [18], associating OGTT and the clamp, demonstrated different levels of IR, insulin hypersecretion, and reduced insulin hepatic clearance in lean and obese hyperinsulinemic PCOS. Results of this study led to speculate that IR and hyperinsulinemia may represent two different metabolic disorders in PCOS: the first depending on obesity, and the second representing a major feature of PCOS. However, the evaluation of insulin secretion after glucose load in PCOS offers different results in respect to clamp and HOMA, mostly in normal-weight subjects [9,21]. In this study, lean PCOS resulted in hyperinsulinemic in response to OGTT in 44% of cases, whereas HOMA-detected IR was found only in 14% of the same subjects. In this group, the metabolic alteration was mainly due to a disproportional rise of pancreatic secretion and reduced hepatic clearance. Recently, these results were partially confirmed by Moghetti et al. [18], who demonstrated that insulin clearance is reduced in all PCOS subjects, which contributed to generating hyperinsulinemia and hyperandrogenism in lean subjects. Moreover, in a recent study, a failure of HOMA to identify about 50% of lean PCOS subjects with clamp-detected IR was reported [28].

Many young subjects may have benefits from early-onset diagnosis and treatment, but a proper diagnosis of metabolic problems is not currently available. Recent findings determined that the early treatment of insulin resistance and hyperandrogenism can provide a diminution of hepato-visceral adiposity, leading to restore ovulation and reducing the risk of diabetes, the main metabolic complication [27]. Therefore, early detection and control of insulin metabolism should be an important aspect of preventive medicine in adolescent and young PCOS.

Metabolomics is a promising tool for global endogenous metabolite analysis in biological systems and has been widely applied to the study of several pathologies, the discovery of new biomarkers, and better understanding of biochemical pathways associated with disease development [29,30]. Metabolites are the end product of biochemical reactions in the body; therefore, they are the closest molecules to phenotype. Thus, metabolomics can be a promising tool to the potential identification of biomarkers of an excessive insulin response to OGTT and may represent a significant progress regarding the prevention, diagnosis, and treatment of PCOS.

In this study, a ^1^H-NMR coupled multivariate statistical analysis metabolomics approach was performed in patients with PCOS to identify a panel of urinary biomarkers of hyperinsulinemia and insulin resistance.

The aim of this study was to identify a possible yet noninvasive method to properly evaluate insulin metabolism in young nondiabetic subjects.

## 2. Results

Table 1 illustrates the anthropometric, hormonal, metabolic, and US characteristics of all subjects and in study groups in relation to BMI. Age, glycemic values, and hormonal parameters are similar in the two groups, whereas the insulin and the HOMA values are increased in the obese subjects.

None of the subjects had diabetes, but the sample shows a high percentage of familiarity with diabetes (59.52%).

Applying the previously mentioned parameters, patients were classified as insulin sensitive (IS) or insulin resistant (IR) and as normo-insulinemic (NI) or hyperinsulinemic (HI). Data of the four subgroups are reported in Table 2.

Based on HOMA results, 15 of the 42 PCOS subjects were classified as IR (35.1%). Considering the I-AUC, 24 subjects were classified as HI (57.14%).

The HOMA index failed to identify IR in 13 of the 24 subjects who displayed hyperinsulinemia by I-AUC, such that the normal HOMA index underestimated 46.42% of cases (53.57% sensitivity, 100% specificity) in patients who exhibited increased insulin secretion. Three overweight or obese PCOS subjects (BMI > 24.99), presented increased HOMA values, resulting in normo-insulinemia under OGTT (26%), such that, in our group, the increased HOMA index potentially led to overestimate 26% of cases.

There were 11 IR PCOS subjects who resulted in hyperinsulinemic following OGTT (73%), showing both insulin metabolism alterations, nine of whom were overweight or obese or both.

### H-NMR Results

**Figure 1:**
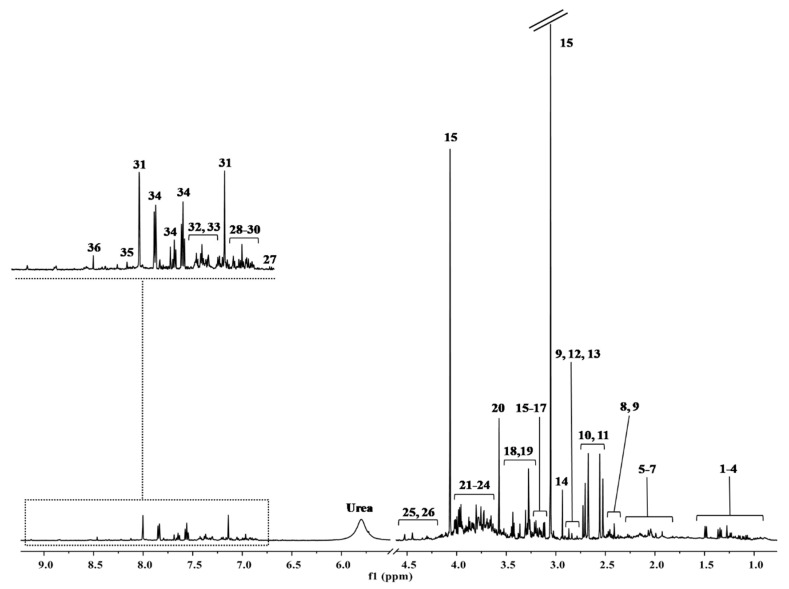
The major spectral area assignments of a representative ^1^H-NMR spectra of urine. Metabolites were identified based on literature information and by using a dedicated library, such as the Human Metabolome Database (HMDB, http://www.hmdb.ca, accessed on 29 March 2020) and the 500 MHz library from Chenomx NMR suite 7.1. **Peaks**: (1) 3-methyl-2-oxovalerate, (2) 2-hydroxyvalerate, (3) isoleucine, (4) valine, (5) 2-aminoadipate, (6) homoserine, (7) glutamine, (8) pyruvate, (9) 3-phenylpropionate, (10) citrate, (11) dimethylamine, (12) trimethylamine, (13) asparagine, (14) dimethylglycine, (15) cis-aconitate, (16) ethanolamine, (17) o-phosphocholine, (18) trimethylamine n-oxide, (19) taurine, (20) glycine, (21) creatine, (22) creatine phosphate, (23) guanidinoacetate, (24) glycolate, (25) threonine, (26) cytidine, (27) 3-hydroxykynurenine, (28) 3-hydroxymandelate, (29) tyrosine, (30) kynurenine, (31) histidine, (32) phenylacetylglycine, (33) tryptophan, (34) hippurate, (35) nicotinic acid, (36) formate.

The representative ^1^H-NMR spectrum of urine samples is shown in Figure 1.

**Figure 2:**
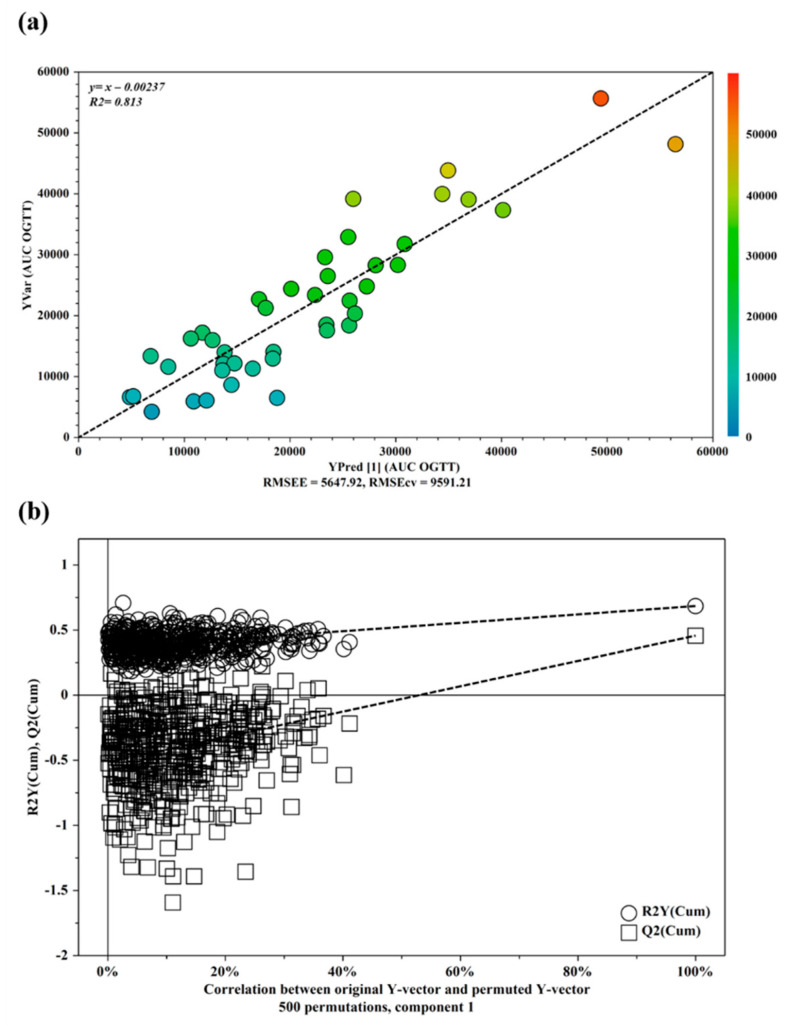
(**a**) OPLS plot showing the relationship between the metabolic profile and the AUC OGTT values. The horizontal axis represents the observed values, and the vertical axis represents the predicted values. (**b**) Validation plots of OPLS model using a permutation test. The horizontal axis shows the correlation between the permuted and actual data, while the vertical axis displays the cumulative values of R^2^ and Q^2^. The intercept gives an estimate of the overfitting phenomenon.

Metabolites were identified based on literature information and by using a dedicated library, such as the Human Metabolome Database (HMDB, http://www.hmdb.ca, accessed on 29 March 2020) and the 500 MHz library from Chenomx NMR suite 7.1. The whole ^1^H-NMR dataset was subjected to multivariate statistical analysis. A first PCA analysis (data not shown) was conducted on the entire dataset in order to highlight potential outliers (outside the 95% confidence limit) due to interfering peaks or instrument errors. Once identified as outliers, due to interference in the sample, three patients were excluded from further statistical analysis. Then, OPLS analysis was applied to samples in order to evaluate the potential relationship between the metabolic profiles (X-variables) and the AUC OGTT values (Y-variables). The OPLS model indicated that the NMR profile of urine had a good fit and prediction ability for the AUC OGTT with R^2^ = 0.813 (Figure 2a).

The OPLS model was established with one predictive and one orthogonal component and showed good values of R^2^X, R^2^Y, and Q^2^ (Table 3). The validity of the OPLS model was evaluated through a permutation test (Figure 2b) used 500 times.

The test results are reported in Table 4 and indicate the statistical validity of the OPLS model. A further OPLS model was built to evaluate the potential correlation between the urinary profile and the HOMA value. The OPLS model was established with one predictive and one orthogonal component and showed a negative Q^2^ value (Q^2^ = −0.253), indicating a bad fit and prediction ability.

**Figure 3:**
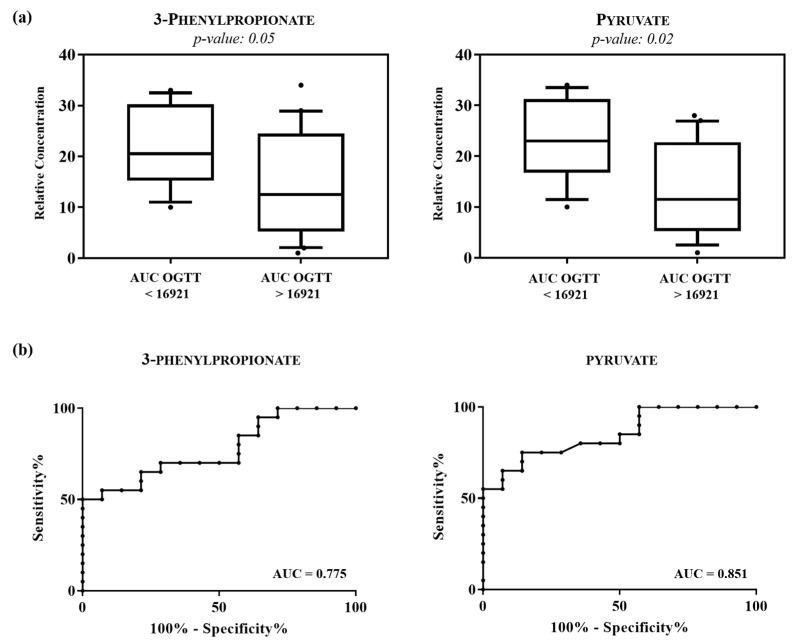
(**a**) Box-and-whisker plots show progressive changes of the urine metabolite levels on subjects with AUC OGTT < 16,921 and AUC OGTT > 16,921. Statistical significance was determined using the Mann-Whitney U test and a *p*-value < 0.05 was considered statistically significant. The Benjamini-Hochberg adjustment was applied. (**b**) Representative ROC curves built for each altered metabolite in AUC OGTT < 16,921 compared with AUC OGTT > 16,921.

The discriminant metabolites based on VIP values of > 1, such as 2-aminoadipate, 3-phenylpropionate, pyruvate and trimethylamine were quantified using Chenomx NMR suite 7.1 and subjected to the Mann-Whitney U test to determine their statistical significance. The results of the univariate statistical analysis showed that only 3-phenylpropionate and pyruvate changed significantly in subjects characterized by AUC OGTT value > 16,921 compared with subjects with value < 16,921 (Table 4a). The relative concentrations of these metabolites in the two groups were compared using box-and-whisker plots. As shown in Figure 3a, subjects with AUC OGGT > 16,921 were characterized by a lower level of 3-phenylpropionate and pyruvate compared with subjects with AUC OGGT value < 16,921. Finally, the individual ROC for each metabolite was built and shown in Figure 3b. The corresponding statistical parameters are reported in Table 4b. The receiver operating characteristic (ROC) curve and the determination of the area under the curve (AUC) is a commonly used method to evaluate the diagnostic potential of a classifier in clinical applications. Subsequently, to evaluate if the model showed a sensitivity and specificity better than the single metabolites, a ROC curve was constructed by using the combination of the two metabolites. The results of the analysis showed an AUC of 0.875 (95% CI: 0.754–1.00), indicating how the combination of the metabolites exhibit an AUC slightly higher compared to the single pyruvate metabolite (Figure S1, supporting information).

## 3. Discussion

The strength of this analysis is that both HOMA and OGTT are used to define peripheral IR and the post-challenge condition of hyperinsulinemia in PCOS subjects and to ascertain the presence of a urine marker for both IR and hyperinsulinemia. The choice to study young PCOS subjects derives from the high possibility that the affected subjects have some form of insulin secretion or function disorder, independent of age and body weight, and in presence of a similar hormonal environment.

The greatest health organizations, such as the Endocrine Society, the American Association of Clinical Endocrinologists, the Androgen Excess and PCOS Society, and the American College of Endocrinology recommend to assess women with PCOS for type 2 diabetes using OGTT, whereas HOMA is suggested to evaluate IR in obese subjects [14].

Nevertheless, recent studies suggest that hyperinsulinemia, due to increased β-cell secretory capacity, is a prominent feature in PCOS subjects independent from body weight, which drives levels of androgen excess in PCOS [16,28,31].

It is important to evaluate the degree of hyperinsulinemia, in part because it increases to compensate for insulin resistance [31], and because it is related to the primary alteration in insulin liver catabolism [18]. Therefore it can represent an important risk, and help to determine cardiometabolic disease in the affected population [31].

Addressing disparities in IR and hyperinsulinemia in normal-weight and obese PCOS can be an important and new approach to the diagnosis and management of metabolic dysfunction and provide further insights into the metabolic disease risks of all PCOS subjects. Our study population was very young, and it represented an advantage, because all metabolic problems related to the insufficient pancreatic insulin production and diabetes onset were reduced.

In the present study, we sought to find a urinary marker for both measures of insulin sensitivity and in the post-challenge response of insulin in nondiabetic women with PCOS.

Data confirmed previous results on the presence of different kinds of metabolic alterations in PCOS subjects, often not detectable by HOMA, representing an indication for 180 min OGTT.

The finding that urine had reduced amounts of 3-phenylpropionate and pyruvate may indicate a new way to diagnose hyperinsulinemia in PCOS subjects.

The data obtained shows that there is a correlation between AUC values and the urinary metabolomics profile of the patients with PCOS. The multivariate statistical analysis has highlighted significant differences of the metabolomics profile between hyperinsulinemic subjects (AUC >16,921) compared with noninsulin resistant subjects. The hyperinsulinemic subjects showed significantly altered levels of 3-phenylpropionate and pyruvate with respect to noninsulin resistant subjects. 3-phenylpropionate is a metabolite derived from gut microbial metabolism and metabolized by the gut microbiota, E. coli, which transforms it into 2-hydroxypenta-2,4-dieneoate and succinate [32,33]. Different studies report that the alterations in gut microbiota are associated with PCOS and insulin resistance [34,35]. The androgen level in women with PCOS is always high and studies on animal models report that the composition of the microbiota at the time of puberty is strongly influenced by sex hormones [35]. In particular, specific changes in gut microbiota, due to enrichment in Gram-negative species, result in a greater translocation of some molecules (such as lipopolysaccharide (the most potent inflammatory trigger), produced by Gram-negative bacteria) through the intestine, resulting in a chronic state of low-grade inflammation. By binding to the Toll-like receptor-4 (TLR-4)–CD14 complex, Lipopolysaccharide (LPS) activates the immune system, interfering with the insulin receptor and leading to an increase in testosterone production resulting in PCOS and insulin resistance [36,37].

Further studies indicate how gut dysbiosis in subjects with POCS leads to the production of metabolites capable of regulating the secretion of brain-intestine peptides and islet β-cell proliferation, thus leading to an accumulation of abnormal fat, insulin resistance, and hyperinsulinemia [38]. A study by Rui Liu et al. [39] reported that the genera Bacteroides and Escherichia/Shigella are significantly altered in the intestine of women with PCOS, indicating a potential correlation between gut microbiota and the development of metabolic disorders in PCOS.

Data analyses show how the hyperinsulinemic subjects were characterized by significantly altered levels of pyruvate in respect to noninsulin resistant subjects. Pyruvic acid is a key metabolite of cellular metabolism and constitutes the final product of glycolysis. Subsequently, pyruvate is transformed by the pyruvate dehydrogenase enzyme into Acetyl-CoA, which enters the Krebs cycle. The alteration of pyruvic acid and the related pathways is well known in the literature in subjects with PCOS and IR [40,41,42,43]. Metabolomics studies conducted on follicular fluid in women with PCOS indicate reduced glucose availability in oocytes and follicular cells caused by faulty glucose transport in PCOS [40,43]. Physiologically, in mature oocyte cells, the transport of glucose is insulin dependent through type 4 glucose transporters (GLUT-4). In PCOS and hyperinsulinism, the expression of GLUT-4 is suppressed [44]. The decreased availability of glucose in the oocytes and in the follicular cells, caused by the faulty transportation of glucose, determines the use of alternative energy pathways. These compensation mechanisms are reflected in the altered level of different molecules, such as pyruvate, lactate, lipids and amino acids, in subjects with PCOS and hyperinsulinism compared with healthy subjects [40]. Indeed, Yan Zhang et al., in a metabolomics study conducted on follicular fluid samples of 15 PCOS subjects compared with 36 control subjects, reported an increase in ketone bodies, such as acetoacetate and 3-hydroxybutyrate, in the follicular fluids of individuals with PCOS. Acetoacetate and 3-hydroxybutyrate originate from the catabolism of fatty acids. The subjects with PCOS and hyperinsulinism showed an upregulation of the degradation of fatty acids in order to potentially compensate for the impairment of pyruvate metabolism and glycolysis in energy demands. Furthermore, the impairment of the Krebs cycle is also reported in a metabolomic study conducted on the urinary profile of subjects with PCOS compared with a control group [41]. The authors highlighted a reduction of 2-oxoglutarate and isocitrate as well as sugars involved in the TCA cycle, such as xylose and d-allose, in the urinary profile of subjects with PCOS, indicating impairment of the TCA cycle. Finally, metabolic studies conducted on the plasma of subjects with PCOS and hyperinsulinism indicate the alteration of various plasma metabolites such as citrate, confirming that subjects affected by PCOS and hyperinsulinemia were characterized by an impairment of the Krebs cycle and that the demand for energy was satisfied through alternative routes [45,46]

## 4. Materials and Methods

### 4.1. Study Population (Pilot Study)

A total of 250 Patients aged 14–22 years, afferent to the center of Gynecological Diseases in Childhood and Adolescence of the Policlinico Universitario Duilio Casula, Monserrato (Università degli Studi di Cagliari), Italy, between January 2018 and December 2019, were screened for PCOS, following Carmina criteria in adolescence. Out of a total of 113 subjects presenting irregular menstrual cycles after 2 years from menarche, and the availability to perform hormonal and metabolic evaluation and pelvic ultrasound, 60 subjects were excluded because of technical limits of the pelvic US examination, 7 subjects were excluded after the finding of ovarian cysts (> 3 cm of diameter), and 4 subjects refused to participate to the study protocol, therefore, urine sampling was obtained from 42 girls in total, and all subjects and parents accepted to partake in the study. The presence of clinical hyperandrogenism was assessed. Hirsutism was evaluated using the mFG (modified Ferriman Gallwey) score [47], including nine body regions, using a score range from zero (absence of terminal hairs) to four (extensive terminal hair growth). Evaluation of acne was performed using the Cremoncini scale [48]. Dysmenorrhea was assessed with the visual analogue scale (VAS) [49]. Body mass index (BMI) was calculated as the ratio between weight and height. WHR (waist-hip ratio) was calculated as the ratio between waist and hip circumferences.

**Figure 4:**
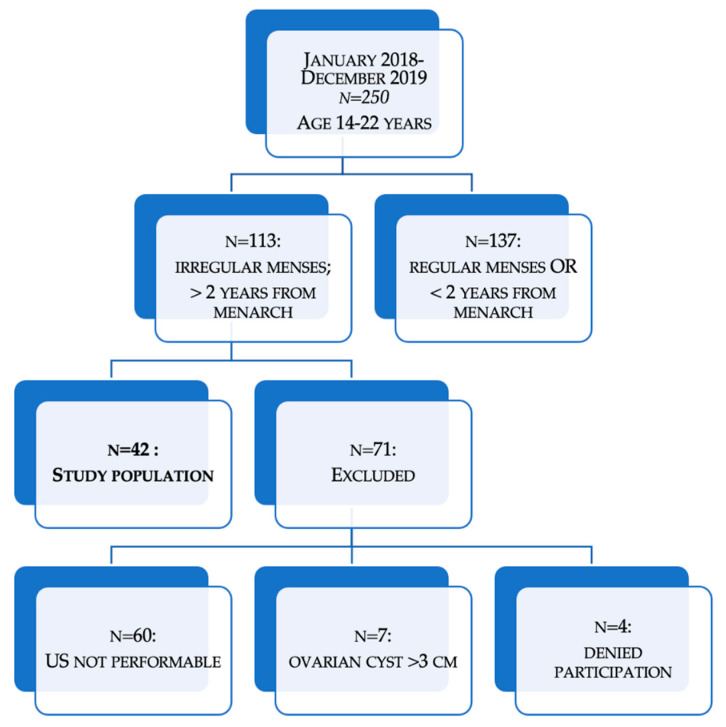
Flow chart of study population selection.

Figure 4 shows a flow chart diagram, which indicates the study population.

PCOS was diagnosed according to Rotterdam Criteria, and adapted to adolescence from Carmina [50]. In patients presenting, for at least 2 years after menarche, the following criteria: hyperandrogenemia either associated or not to biochemical signs of hyperandrogenism, chronic anovulation or oligo-amenorrhea or both, and polycystic ovaries at ultrasound, including ovarian size greater than 10 cm^3^.

### 4.2. Study Protocol

Hormonal assays, OGTT, transabdominal (TA) or transvaginal (TV) US, and urine collection were assayed in the early follicular phase. In patients who had secondary amenorrhea, menstruation was induced with an oral intake of 10 mg of Medroxyprogesterone acetate for 5 days.

All subjects underwent blood sampling under fasting conditions, blood was centrifuged, and serum was stored at −20 °C until assayed. Hormonal assays included FSH, LH, 17-ßestradiol (E2), total testosterone (tT), delta-4-androstenedione (A), 17-hydroxyprogesterone (17-OHP), and prolactin (Prl).

Patients were subjected to a 75 g OGTT (Oral Glucose Tolerance Test), analyzing glycemia and insulinemia after oral glucose load at minute 0′, 30′, 60′, 90′, 120′, and 180′.

The ultrasonographic examination was performed the same day, using a high-performance ultrasound machine (Voluson I, General Electric, Milwaukee, WI, USA). A convex TA probe (3.5–5 MHz) was used for a transabdominal exam and a 5–9 MHz frequency transvaginal probe was used for pelvic US. TV approach was performed in subjects who were sexually active.

An ovarian volume equal to or above 10 cm^3^, according to Rotterdam criteria, was considered a significant threshold for PCOS, and it was calculated with a two-dimensional (2D) scan using the following formula: $\frac{\pi }{6}$ × (A × B × C), where “A” represents the length, “B” the width and “C” the thickness of the ovary.

The ovarian maximal plain section was used to evaluate the follicle distribution pattern and to count the follicle number per single cross-section (FNPS), which considers a threshold equal to or above 12 follicles significant for PCOS.

The following day, a urine sample was collected from each patient in the afternoon after 4 h from the end of standardized lunch with 80 gr of pasta, salad, and fruit [51] and immediately stored at −80 °C, until assay.

### 4.3. Assays

Serum LH, FSH, E2 and tT were assayed by chemiluminescence immunoassay, (SIEMENS Immulite USA SIEMENS Products Corporation, Los Angeles, CA, USA).

Radioimmunoassay was used to measure serum Prl, A, and 17-OHP (Diagnostic 151 System Laboratories, Inc. DSL, Webster, TX, USA).

Assays of A cross reacted principally with androsterone and with T (0.58% and 0.24% respectively); tT cross reacted with 5 alfa-dihydrotestosterone and with A (2% and 0.6%, respectively); 17OHP cross reacted with 17OH pregnenolone, progesterone, and prednisone (4.1%, 1.3%, and 0.23%, respectively); LH cross reacted principally with HCG (0.2%); and Prl cross reacted with the other hormones (<0.5%).

The intra-assay and interassay coefficients of variation at low medium and high concentrations for each of the analytes were as follows: LH: 13.0% and 23.9% for low level, 3.6% and 6.7% for medium level, 6.0% and 7.1% for high level, respectively; FSH: 4.9% and 4.1% for low level, 3.2% and 4.1% for medium level, and 3.1% and 7.9% for high level, respectively; E2: 11.0% and 2.0% for low level, 2.7% and 2.0% for medium level, and 2.6% and 0.9% for high level, respectively; tT: 16.3% and 24.3% for level low, 15.2% and 15.6% for medium level, and 5.1% and 7.2% for high level, respectively; A: 4.5% and 9.0% for low level, and 3.7% and 5.9% for medium level, respectively; Prl: 5.1% and 5.8% for low level, 3.9% and 4.8% for medium level, and 3.4% and 5.4% for level high, respectively; 17OHP: < 7% and 1.0% for level low, < 7% and 14.9% for medium level, and < 7% and 8.7% for high level, respectively.

Cut-off levels were evaluated inhouse and were previously utilized as reference for normal levels [9].

Insulin and glucose serum values were analyzed in all samples after OGTT and were measured by RIA (Diagnostic 172 Systems Laboratories, Inc. [DSL], Webster, TX, USA).

The lower limit of detection for analytes in our lab was: FSH and LH: 0.1 mIU/mL; tT: 0.5 nmol/L; A: 0.3 ng/mL; 17OHP: 0.3 ng/mL; E2: 10 pg/mL.; Prl 0.35 ng/mL.

### 4.4. Urine Samples Preparation

800 µL of urine mixed with 8 µL in a 1% aqueous solution of NaN3, to inhibit bacteria growth, was transferred into an Eppendorf tube and stored at −80 °C. Before the analysis, the sample was centrifuged at 12,000× *g* for 10 min at 4 °C. Then, 630 µL of the supernatant was mixed with 70 µL of potassium phosphate buffer in D2O (1.5 M, pH 7.4) containing sodium 3-trimethylsilyl-propionate-2,2,3,3,-d4 (TSP) as an internal standard (98 atom% D, Sigma-Aldrich, Milan, Italy). A quantity of 650 µL was transferred to 5-mm NMR glass tubes for ^1^H-NMR analysis [52].

### 4.5. ^1^H-NMR Spectroscopic Analysis

NMR analysis was carried out using a Varian UNITY INOVA 500 spectrometer operating at 499.839 MHz for protons and equipped with a 5 mm double resonance probe (Agilent Technologies, CA, USA). The acquisition parameters of the ^1^H-NMR spectra are reported in our previous article [52]. The FIDs were weighted by an exponential function with a 0.5-Hz line-broadening factor prior to Fourier transformation.

### 4.6. NMR Data Preprocessing

NMR spectra were phased, and baseline corrected using an ACDlab Processor Academic Edition (Advanced Chemistry Development, 12.01, 2010) and chemical shifts referenced to an internal standard at δ = 0.0 ppm. The spectral region comprising the signal of residual water and urea (4.7–6.5 ppm) was removed. The ACD Labs intelligent bucketing method was used for spectral integration [53]. A 0.04 ppm bucket width was defined with an allowed 50% looseness. The area of bucketed regions was normalized using Median Fold Change Normalization (MFC) [54]. Finally, the spectral data were imported into the SIMCA software (Version 15.0, Sartorius Stedim Biotech, Umea, Sweden) for statistical multivariate analysis. All imported data were then preprocessed using Pareto scaling. Pareto scaling increases the representation of lower concentration metabolites in the resultant data models, while minimizing noise contribution.

### 4.7. Data Analysis

Hyperprolactinemia was diagnosed when prolactin levels were >20 ng/mL (>69.8 nmol/L). Hyperandrogenism was defined as total testosterone (tT) at >0.7 ng/mL (>2.43 nmol/L), and 17-hydroxyprogesterone (17OHP) at >1.4 ng/mL, (>4.45 nmol/L), and delta-4-androstenedione (A) at >3.5 ng/mL (>12.2 nmol/L). These cut-offs were previously validated in adult women with the use of assays established in our laboratory [55]

A normal response to OGTT was defined for glycemia, according to the criteria of the National Diabetes Data Group [56]. Insulin and glucose after glucose oral load were expressed as the area under the curve (I-AUC), calculated according to the trapezoidal rule. The definition of hyperinsulinemia in our population was previously obtained in a healthy group of young subjects with the same ethnicity, using the mean 2 + SD as previously described [9,11,21]. The test was considered normal when I-AUC was < 16,921 mIU/mL after 180 min [9].

The HOMA was calculated by the multiplication of the values of fasting glucose (mmol/L) and insulin (mU/mL) and divided by a constant $\left[(I^{0}\times G^{0})÷22.5\right]$. Based on the 95% confidence levels, the HOMA cut-off obtained in our previous study was 3.6 [9], whereas the best normal cut-off in adolescence reported from a recent metanalysis was reported between 2.3 and 3.6 [57], therefore, we used 3.6.

### 4.8. Multivariate Statistical Analysis

For multivariate statistical analyses of ^1^H-NMR data, two different procedures were employed: principal component analysis (PCA) and orthogonal partial least square (OPLS). PCA was performed on the spectral data set in order to evaluate the homogeneity of the samples and identify any possible trends and outliers between the samples [58]. OPLS removes the systematic variation in X (peak intensity in NMR spectra) that is orthogonal to Y (i.e., dichotomous and continuous variables) [59]. In this work, OPLS regression analysis was used to investigate the relationship between the AUC OGTT value and metabolomics profile of the patients. The quality of the OPLS model was evaluated using a 7-fold cross-validation and permutation test (500 times). The permutation plot displays the correlation coefficient between the original *y*-variable and the permuted *y*-variable on the *x*-axis versus the cumulative R^2^ and Q^2^ on the *y*-axis and draws the regression line. The intercept is a measure of the overfit. A Q^2^Y intercept value less than 0.05 is indicative of a valid model. The estimated predictive power of the models was expressed by R^2^Y and Q^2^Y, which represents the fraction of the variation of *y*-variable and the predicted fraction of the variation of *y*-variable, respectively. A good prediction model is achieved when Q^2^ ≥ 0.5.

### 4.9. Univariate Statistical Analysis

The major variables were obtained by evaluating a VIP (Variables Important Projection) value greater than 1 and were quantified using the Chenomx NMR Suite 7.1 (Chenomx Inc., Edmonton, AB, Canada) library [60]. GraphPad Prism software (version 7.01, GraphPad Software, Inc., San Diego, CA, USA) was used to perform the univariate statistical analysis of the data. Statistical significance was assessed using the Mann-Whitney U test and a *p*-value of < 0.05 was considered statistically significant. The Benjamini-Hochberg adjustment was subsequently applied to the obtained *p*-values to acquire the level of significance for multiple testing [61]. The diagnostic power of the potential biomarkers was assessed by constructing a receiver operating characteristic (ROC). The GraphPad Prism software was used to generate ROC curves and calculate sensitivity, specificity, and the area under the ROC curve (AUC).

## 5. Conclusions

Our data showed a high significant correlation between AUC and urine NMR profiles, and significant urinary phenylpropionate and pyruvate reduction after a meal in hyperinsulinemic subjects.

This is new information in a pilot study on altered metabolism after a meal in hyperinsulinemic subjects, which is absent in IR subjects.

On the contrary, IR subjects do not exhibit specific significant alteration of urine metabolites compared to noninsulin resistant subjects.

Based on this study, measuring 3-phenylpropionate and pyruvate may be a useful and fast tool to detect insulin sensitivity in nondiabetic women with PCOS.

Further studies and larger sample sizes will be useful to better clarify the clinical importance of this metabolic aspect.

## Figures and Tables

**Table 1:** Anthropometric, hormonal and metabolic characteristics of all patients and in two subgroups in relation to BMI. Values are mean ± SD.

		BMI 16–24.99	BMI 25–41	*p-*Value
N. of patients	42	20	22	
Age	18.85 ± 4.45	18.85 ± 4.78	18.86 ± 4.23	NS
BMI (kg/m^2^)	26.33 ± 5.94	21.42 ± 2.21	30.61 ± 4.67	<0.001
Ferriman & Gallwey score	12.48 ± 5.36	12.15± 5.48	12.77 ± 5.36	NS
Cremoncini score	1.36 ± 1.39	1.55 ± 1.54	1.18 ± 1.26	NS
Dysmenorrhea (VAS)	2.74 ± 2.98	3.3 ± 3.45	2.23 ± 2.45	NS
Familiarity with diabetes (%)	59.52%	40%	77.27%	0.01
Glycaemia (mmol/L)	4.55 ± 0.52	4.47 ± 0.68	4.62 ± 0.32	NS
Insulin (pmol/L)	119.13 ± 57.5	96.31 ± 43.97	140.04 ± 61.32	0.01
HOMA	3.49 ± 1.77	2.76 ± 1.34	4.16 ± 1.87	0.01
AUC (pmol/L)	149,019.81 ± 87,376.84	113,639.8 ± 63,511.19	181,388.27 ± 94,805.59	0.01
Insulin 120′ (pmol/L)	1003.76 ± 638.25	736.5 ± 462.49	1276.03 ± 675.18	<0.01
Insulin 180′ (pmol/L)	564.12 ± 504.24	391.03 ± 376.81	723.02 ± 560.71	0.03
Insulin—max. value (pmol/L)	1289.52 ± 782.94	1051.18 ± 652.52	1507.97 ± 842.15	NS
Glyc. 120′ (mmol/L)	6.31 ± 1.33	5.92 ± 1.11	6.63 ± 1.43	NS
Glyc 180′ (mmol/L)	5.65 ± 1.8	5.47 ± 1.68	5.81 ± 1.93	NS
Total cholesterol (nmol/L)	4.36 ± 0.9	4.4 ± 1.02	4.33 ± 0.75	NS
HDL (nmol/L)	1.49 ± 0.46	1.54 ± 0.44	1.42 ± 0.48	NS
LDL (nmol/L)	2.51 ± 0.7	2.75 ± 0.68	2.2 ± 0.65	NS
Triglyceride (nmol/L)	0.82 ± 0.4	0.76 ± 0.26	0.9 ± 0.52	NS
FSH (Ul/L)	5.51 ± 1.86	5.66 ± 1.99	5.38 ± 1.78	NS
LH (UI/L)	6.84 ± 7.72	5.59 ± 3.54	7.92 ± 10	NS
E2 (pmol/l)	157.77 ± 114.23	133.99 ± 74.7	179.39 ± 139.29	NS
T (nmol/l)	1.54 ± 0.92	1.44 ± 0.83	1.63 ± 1.01	NS
A (nmol/l)	10.58 ± 5.15	9.77 ± 3.98	11.31 ± 6.02	NS
PRL (mg/mL)	13.98 ± 7.56	14.38 ± 8.99	13.59 ± 6.09	NS
17-OHP (nmol/L)	2.75 ± 1.8	2.47 ± 1.61	3.03 ± 1.97	NS
SHBG (nmol/L)	54.44 ± 21.22	61.84 ± 18.99	44.39 ± 20.5	0.02
AMH (pmol/L)	60.34 ± 35.18	59.78 ± 30.97	61.13 ± 41.66	NS
Ovarian volume (mm^3^)	6084.16 ± 2753.42	5515.08 ± 2302.23	7080.04 ± 3334.58	NS
N. of ovarian follicles	11.42 ± 5.28	11.33 ± 4.5	11.54 ± 6.41	NS

**Table 2:** Anthropometric, hormonal, and metabolic characteristics in relation to insulin sensibility and secretion. Values are mean ± SD.

	Insulin Sensibility			Insulin Secretion		
	HOMA ≤ 3.6	HOMA > 3.6	*p*-Value	AUC ≤ 16,921	AUC > 16,921	*p*-Value
N. of patients	27	15		18	24	
Age	19.19 ± 4.7	18.21 ± 4.02	NS	19.94 ± 4.52	18.08 ± 4.33	NS
BMI (kg/m^2^)	24.2 ± 4.22	30.16 ± 6.76	<0.01	24.06 ± 3.29	28.03 ± 6.92	0.03
Glycaemia (mmol/L)	4.49 ± 0.6	4.65 ± 0.33	NS	4.45 ± 0.71	4.62 ± 0.31	NS
Insulin (pmol/L)	83.59 ± 18.18	183.09 ± 47.41	<0.01	101.92 ± 52.01	132.03 ± 59.06	NS
HOMA	2.41 ± 0.63	5.45 ± 1.43	<0.01	2.92 ± 1.6	3.92 ± 1.8	NS
AUC (pmol/L)	135,600.04 ± 85,392.28	173,175.38 ± 88,568.16	NS	73,130.77 ± 25,959.05	205,936.58 ± 72,277.93	<0.01
Insulin 120′ (pmol/L)	907.18 ± 597.45	1218.54 ± 678.63	NS	467.36 ± 196.42	1431.64 ± 529.28	<0.01
Insulin 180′ (pmol/L)	496.19 ± 488.56	686.38 ± 525.76	NS	283.47 ± 163.44	774.6 ± 570.64	<0.01
Insulin—max. value (pmol/L)	1260.06 ± 873.65	1342.56 ± 610.75	NS	657.48 ± 295.72	1763.55 ± 693.54	<0.01
Glyc. 120′ (mmol/L)	6 ± 1.12	6.87 ± 1.53	NS	5.77 ± 1.01	6.66 ± 1.41	0.05
Glyc. 180′ (mmol/L)	5.42 ± 1.9	6.11 ± 1.57	NS	5.69 ± 2.23	5.63 ± 1.51	NS
Total cholesterol (nmol/L)	4.34 ± 0.8	4.42 ± 1.14	NS	4.31 ± 0.85	4.41 ± 0.95	NS
HDL (nmol/L)	1.54 ± 0.47	1.37 ± 0.42	NS	1.47 ± 0.41	1.5 ± 0.5	NS
LDL (nmol/L)	2.63 ± 0.65	2.25 ± 0.79	NS	2.36 ± 0.41	2.6 ± 0.84	NS
Triglyceride (nmol/L)	0.8 ± 0.36	0.87 ± 0.51	NS	0.87 ± 0.48	0.78 ± 0.34	NS
FSH (Ul/L)	5.59 ± 1.76	5.36 ± 2.08	NS	5.46 ± 1.38	5.54 ± 2.17	NS
LH (UI/L)	6.16 ± 4.88	8.02 ± 11.19	NS	5.07 ± 3.54	8.1 ± 9.53	NS
E2 (pmol/l)	151.68 ± 93.86	168.73 ± 147.15	NS	126.78 ± 76	181.01 ± 132.96	NS
T (nmol/l)	1.42 ± 0.78	1.76 ± 1.14	NS	1.54 ± 0.87	1.54 ± 0.98	NS
A (nmol/l)	9.59 ± 3.54	12.58 ± 7.18	NS	9.69 ± 4.33	11.3 ± 5.73	NS
PRL (mg/mL)	14.57 ± 8.38	12.94 ± 5.99	NS	16.32 ± 9.19	12.14 ± 5.53	NS
17-OHP (nmol/L)	2.69 ± 1.85	2.86 ± 1.76	NS	2.78 ± 1.74	2.72 ± 1.9	NS
SHBG (nmol/L)	55.58 ± 21.51	51.82 ± 21.44	NS	63.24 ± 18.96	47.11 ± 20.65	0.03
AMH (pmol/L)	63.35 ± 35.22	54.02 ± 36.11	NS	75.47 ± 38.08	50.79 ± 30.47	NS
Ovarian volume (mm^3^)	5715.4 ± 2300.42	7067.51 ± 3790.79	NS	7522.75 ± 3294.87	5262.1 ± 2097.55	NS
N. of ovarian follicles	9.95 ± 2.7	13.75 ± 7.39	0.05	11.83 ± 5.59	11.16 ± 5.22	NS

**Table 3:** Parameters OPLS model.

	OPLS Models				Permutation (500 Times) *	
	Components ^a^	R^2^Xcum ^b^	R^2^Ycum ^c^	Q^2^cum ^d^	R^2^ Intercept	Q^2^ Intercept
OPLS model	1P + 1O	0.216	0.684	0.500	0.362	−0.516

^a^ The number of Predictive and Orthogonal components used to create the statistical models. ^b,c^ R^2^X and R^2^Y indicates the cumulative explained fraction of the variation of the X block and Y block for the extracted components. ^d^ Q^2^ cum values indicate cumulative predicted fraction of the variation of the Y block for the extracted components. * An R^2^ intercept value less than 0.3–0.4 and a Q^2^ intercept value less than 0.05 are indicative of a valid model.

**Table 4:** (**a**) Statistical differences of metabolites characterized by VIP > 1. (**b**) Statistical parameters of the receiving operator characteristic (ROC) curves of the metabolites with *p*-value < 0.05.

(**a**)				
**Metabolites**	**Mean (SD) of Group (mM) ^a^**			***p*-Value ^b^**
	**AUC OGTT < 16,921**	**AUC OGTT > 16,921**		
3-phenylpropionate	1.945 ± 0.59	1.443 ± 0.71		0.05
Pyruvate	3.489 ± 1.21	1.462 ± 0.57		0.02
(**b**)				
	**AUC**	**Standard Error**	**Confidence Interval 95%**	***p*-Value**
3-phenylpropionate	0.775	0.07	0.619–0.930	<0.01
Pyruvate	0.851	0.06	0.726–0.977	<0.01

Metabolites were selected on VIP > 1 based on OPLS analysis. ^a^ Relative concentrations were calculated by normalization of the molar concentration of each metabolite to the total molar concentration of all four metabolites for each sample. ^b^ Statistical significance was determined using the Mann-Whitney U test and a *p*-value < 0.05 was considered statistically significant. The Benjamini-Hochberg adjustment was applied.

## Data Availability

The data presented in this study are available on request from the corresponding author. The data are not publicly available due to privacy restrictions.

## Supplementary Materials

The following are available online at https://www.mdpi.com/article/10.3390/metabo11070437/s1, Figure S1: Representative ROC curve built by combining all significantly altered metabolites between the two groups.
