# Supplementary material for: Urinary Metabolites Reveal Hyperinsulinemia and Insulin Resistance in Polycystic Ovarian Syndrome (PCOS)
Source: Metabolites. 2021 Jul 2;11(7):437. doi: 10.3390/metabo11070437 (PMC8307496; doi:10.3390/metabo11070437)
Supplement: Supplementary file 1 [file metabolites-11-00437-s001.zip › metabolites-1281879-supplementary.pdf]

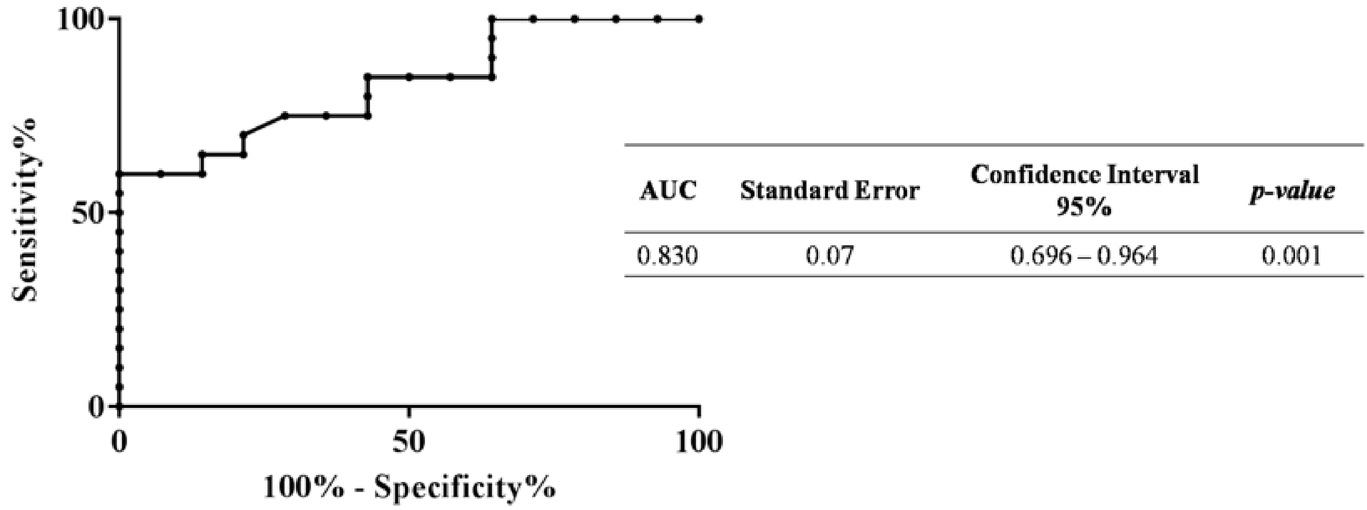

AUC	Standard Error	Confidence Interval 95%	*p-value*
0.830	0.07	0.696 – 0.964	0.001

Figure S1. Representative ROC curve built by combining all significantly altered metabolites between the two groups.
